# Potential therapeutic targets for membranous nephropathy: proteome-wide Mendelian randomization and colocalization analysis

**DOI:** 10.3389/fimmu.2024.1342912

**Published:** 2024-04-19

**Authors:** Zhihang Su, Qijun Wan

**Affiliations:** Department of Nephrology, Shenzhen Second People’s Hospital, The First Affiliated Hospital of Shenzhen University, Shenzhen, China

**Keywords:** membranous nephropathy, Mendelian randomization, plasma protein, therapeutic targets, genome-wide association study (GWAS)

## Abstract

**Background:**

The currently available medications for treating membranous nephropathy (MN) still have unsatisfactory efficacy in inhibiting disease recurrence, slowing down its progression, and even halting the development of end-stage renal disease. There is still a need to develop novel drugs targeting MN.

**Methods:**

We utilized summary statistics of MN from the Kiryluk Lab and obtained plasma protein data from Zheng et al. We performed a Bidirectional Mendelian randomization analysis, HEIDI test, mediation analysis, Bayesian colocalization, phenotype scanning, drug bank analysis, and protein-protein interaction network.

**Results:**

The Mendelian randomization analysis uncovered 8 distinct proteins associated with MN after multiple false discovery rate corrections. Proteins related to an increased risk of MN in plasma include ABO [(Histo-Blood Group Abo System Transferase) (WR OR = 1.12, 95%CI:1.05-1.19, FDR=0.09, PPH4 = 0.79)], VWF [(Von Willebrand Factor) (WR OR = 1.41, 95%CI:1.16-1.72, FDR=0.02, PPH4 = 0.81)] and CD209 [(Cd209 Antigen) (WR OR = 1.19, 95%CI:1.07-1.31, FDR=0.09, PPH4 = 0.78)], and proteins that have a protective effect on MN: HRG [(Histidine-Rich Glycoprotein) (WR OR = 0.84, 95%CI:0.76-0.93, FDR=0.02, PPH4 = 0.80)], CD27 [(Cd27 Antigen) (WR OR = 0.78, 95%CI:0.68-0.90, FDR=0.02, PPH4 = 0.80)], LRPPRC [(Leucine-Rich Ppr Motif-Containing Protein, Mitochondrial) (WR OR = 0.79, 95%CI:0.69-0.91, FDR=0.09, PPH4 = 0.80)], TIMP4 [(Metalloproteinase Inhibitor 4) (WR OR = 0.67, 95%CI:0.53-0.84, FDR=0.09, PPH4 = 0.79)] and MAP2K4 [(Dual Specificity Mitogen-Activated Protein Kinase Kinase 4) (WR OR = 0.82, 95%CI:0.72-0.92, FDR=0.09, PPH4 = 0.80)]. ABO, HRG, and TIMP4 successfully passed the HEIDI test. None of these proteins exhibited a reverse causal relationship. Bayesian colocalization analysis provided evidence that all of them share variants with MN. We identified type 1 diabetes, trunk fat, and asthma as having intermediate effects in these pathways.

**Conclusions:**

Our comprehensive analysis indicates a causal effect of ABO, CD27, VWF, HRG, CD209, LRPPRC, MAP2K4, and TIMP4 at the genetically determined circulating levels on the risk of MN. These proteins can potentially be a promising therapeutic target for the treatment of MN.

## 1 Introduction

Membranous Nephropathy (MN) is a common chronic autoimmune disease. MN may occur in both sexes and all ethnic groups. It is widely acknowledged that MN is the most common cause of adult nephrotic syndrome (1). The prognosis of MN is challenging to predict, as some patients may experience spontaneous remission while others progress to end-stage renal disease (2). Current mainstream treatments for MN include cyclophosphamide, glucocorticoids, rituximab, and calcineurin inhibitors (3). In contrast to newly developed therapies targeting BAFF, plasma cells, and complement (4), human circulating proteins play a crucial role in various biological processes and serve as primary drug targets (5). The relevant literature has shown that protein drug targets with genetic associations supporting their connection to diseases are likelier to become drug targets and gain market recognition than targets discovered by chance (6, 7).

The application of Mendelian randomization (MR) analysis has recently gained significant traction in identifying potential therapeutic targets and repurposing drugs (8). As an approach within genetic instrumental variable analysis, MR employs single nucleotide polymorphisms (SNPs) as instrumental variables (IVs) to estimate the causal relationship between exposures and outcomes (9). Compared to traditional observational studies, MR can mitigate the impacts of type I errors and confounding factors (10).

The association between plasma protein levels and disease risk does not imply a causal relationship. However, suppose there is a specific correlation between genetic variations and protein levels, and these genetic variations are also correlated with disease risk. In that case, it can provide evidence for a causal role of the protein (11). As a result of advancements in large-scale genomic and proteomic technologies, the utilization of MR-based strategies has enabled the discovery of promising targets for therapeutic interventions in a variety of disorders, including Heart Failure (12), multiple sclerosis (13), and inflammatory bowel disease (14). However, no MR study reports have yet integrated MN and protein quantitative trait loci (pQTL) data. In the present investigation, our focus was to ascertain plasma as a viable candidate for therapeutic intervention in the case of MN.

**Figure 1:**
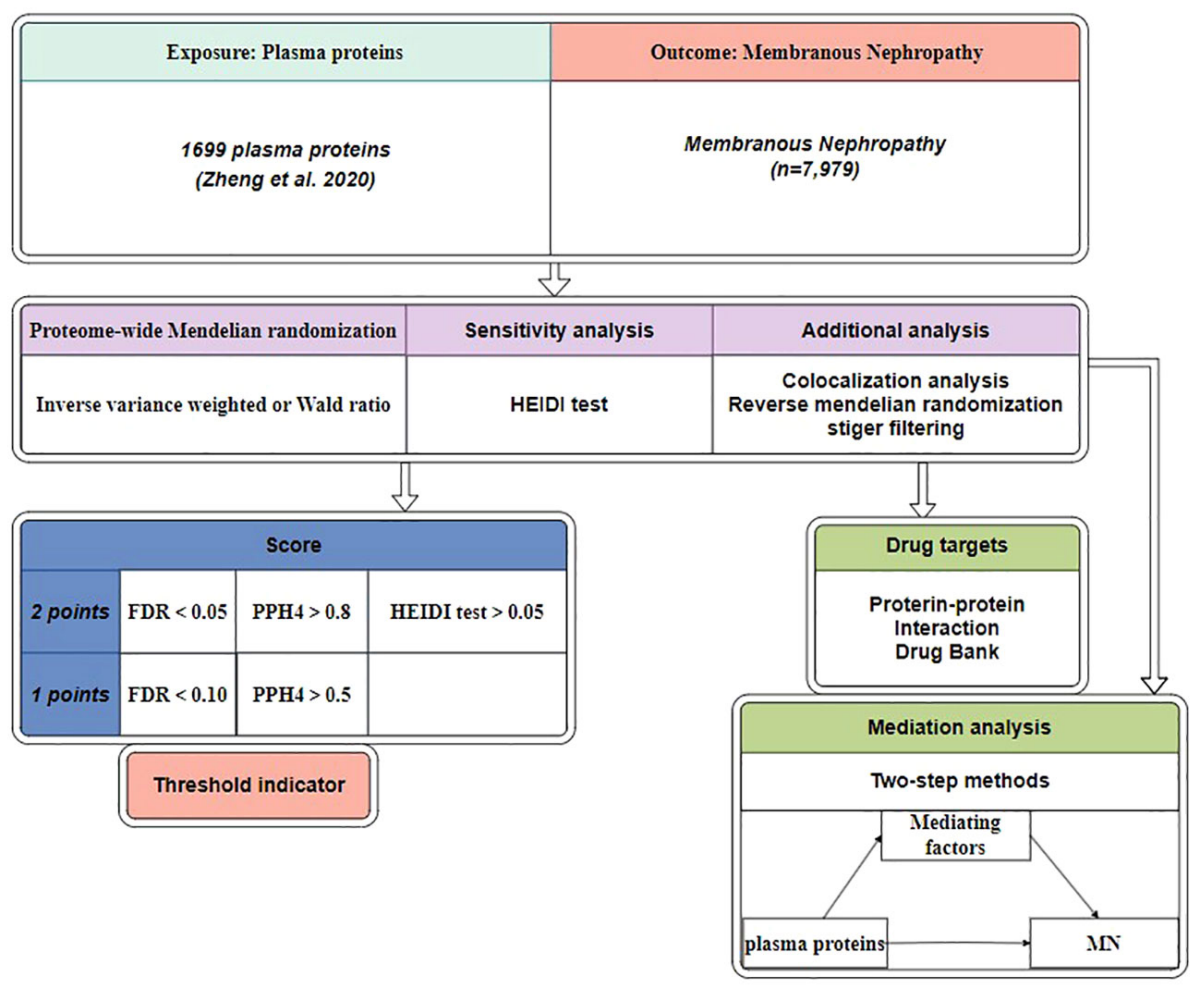
Study design. MN, membranous nephropathy; HEIDI test, heterogeneity in dependent instruments; FDR, false discovery rate; PPH4, posterior probabilities of hypotheses 4.

**Figure 1** depicts the study design. Initially, we utilized MN data from Kiryluk Lab and plasma pQTL data to discern plausible causal plasma proteins associated with MN (15). Next, we further validated the initial findings using Reverse MR, Steiger filtering, Bayesian colocalization analysis, and phenoscanner. We performed a sensitivity analysis using the heterogeneity in dependent instruments (HEIDI) test to examine the robustness of our findings. In a subsequent step, we constructed an interaction network linking potential protein targets with MN drug targets. Subsequently, we evaluated the mediating factors associated with the significant proteins and their impact on MN.

## 2 Methods

This study was conducted according to the reporting guidelines outlined by the Strengthening the Reporting of Observational Studies in Epidemiology (STROBE) initiative (**Supplementary Table 1**).

### 2.1 Plasma protein quantitative trait loci data

We incorporated pQTLs that fulfilled the subsequent set of criteria: ①demonstrating a significant correlation at the genome-wide level (P < 5e-8); ② being situated outside the major histocompatibility complex (MHC) region (chr6, 26-34Mb); ③ demonstrate distinct associations [linkage disequilibrium (LD) clustering r^2 < 0.001]. In the preliminary analysis, we extracted plasma pQTL data from public research (15), which consolidated findings from five general studies (11, 16–19). About the aforementioned selection criteria, a total of 2,113 cis and trans SNPs associated with 1,699 proteins were included (**Supplementary Table 2**). The aforementioned databases have been concurrently utilized in previous studies, alleviating concerns regarding their reliability (13). The above databases are all derived from European populations.

### 2.2 Genome-wide association study data of membranous nephropathy

Aggregate statistical data of MN, including individuals of European ancestry (Sample size of Cases=2150, Sample size of Control=5829, size of SNPs=5,327,688), were retrieved from the Kiryluk Lab (20). All cases included in the final database were diagnosed as idiopathic membranous nephropathy using the gold standard method of renal biopsy. Furthermore, potential causes of secondary membranous nephropathy, such as medication, malignancies, infections, and autoimmune diseases, were systematically ruled out. Nonetheless, the study did not present a detailed description of the target antigens in the patients. Plasma protein and MN come from separate cohorts, indicating no sample overlap between the databases. This suggests that the probability of bias occurring is 0, and the likelihood of a Type I error is 5% (https://sb452.shinyapps.io/overlap/) (21).

### 2.3 Mendelian randomization analysis

In the plasma above protein database, researchers identified genotype-protein associations (pQTL), offering novel insights into the genetic regulation of protein expression. The co-occurrence of pQTL with disease susceptibility loci signifies the molecular consequences of disease-related variations. Previous research has indicated that evidence of the causal role of protein biomarkers in diseases can be revealed through Mendelian randomization analysis, provided that there is an overlap between quantitative trait loci for protein abundance, gene expression, and disease-related loci. Within this study, proteins were investigated as the primary variable of interest, representing the exposure, while MN served as the focal point of analysis, meaning the outcome. The Wald ratio (WR) was utilized for one SNP (22). When two or more SNPs were accessible (the numbers of SNPs≥2), we used the inverse variance weighted (IVW) method (23). In conducting Mendelian randomization (MR), in cases where effect allele frequency data is missing, we employ data from the 1000 Genomes project (HG19/GRCh37) as a substitute. The amplified risk of MN was indicated as odds ratios (OR) per one-unit change in plasma protein levels, corresponding to a standard deviation (SD) increase.

### 2.4 Selection of instrumental variables

**Figure 2:**
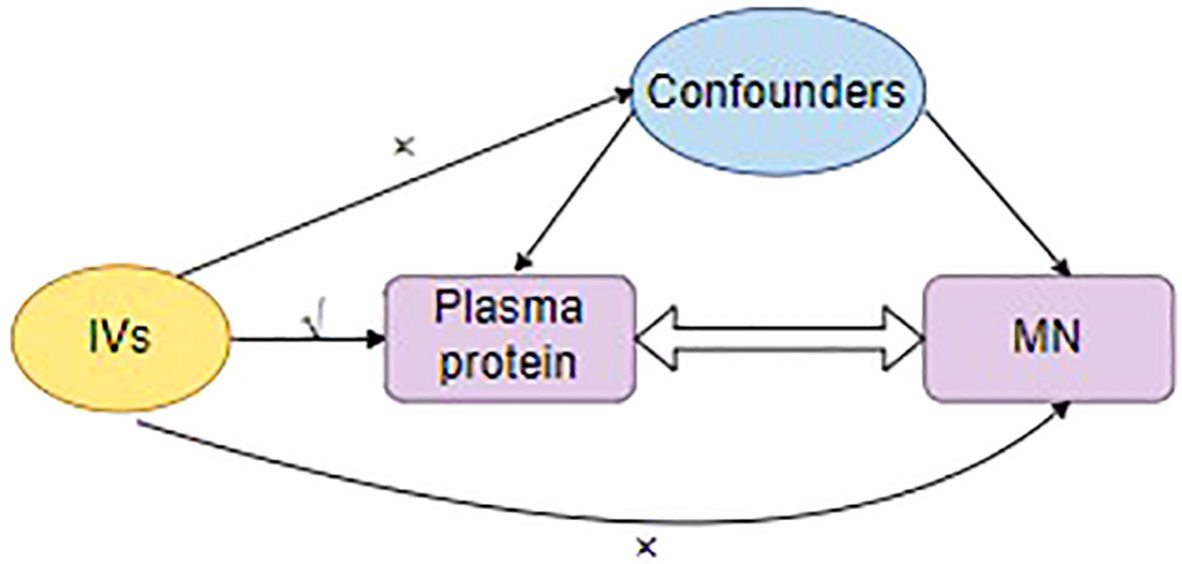
The three major assumptions for the selection of instrumental variables. IVs, Instrumental variants; MN, membranous nephropathy.

The IVs must meet the mentioned assumptions: relevance, exogeneity, and independence (**Figure 2**).

- The IVs must exhibit a strong association with plasma protein. First, we selected SNPs falling beneath the locus-wide significance cutoff (5 × 10^−8^).
- The clumping processes were executed to maintain the IVs’ independence and remove the linkage disequilibrium (LD) effect. Linkage disequilibrium (r²) was set at 0.001, and genetic distance was set at 10,000 kb.
  iii. F = R2 (n - k - 1) /k (1 - R2), where R2 is the exposure variance calculated based on the data within the dataset. The number of IVs is k, and the samples are n. When F-statistic<10, it indicates weak IVs, and they will be excluded.

### 2.5 HEIDI test

We employed the heterogeneity in dependent instruments (HEIDI) test to distinguish between pleiotropy and linkage models (24). Associations with a HEIDI test p-value < 0.05 were considered to be driven by pleiotropy and were thus excluded from further analysis. The SMR software tool (SMR v1.0.3) was used for analysis. Through subsequent HEIDI tests, this approach differentiated potential causal relationships from the genome’s extensive linkage disequilibrium (LD).

### 2.6 Reverse causality detection

Considering MN as exposure and significant protein as the outcome, we will conduct a Reverse MR analysis. We also resort to Stiger filtering as an alternative, a directional testing method (25). Directionality testing involves using Steiger filtering to determine if the SNP correlates more strongly with the outcome than the exposure. SNPs failing this test are considered irrelevant to the exposure and excluded from the analysis (26).

### 2.7 Bayesian colocalization analysis

Colocalization analysis evaluated the likelihood of two traits sharing a common causal variant. As previously mentioned, the posterior probabilities of five hypotheses were utilized to provide detailed supplementation to the results of colocalization analysis. Specifically, the first hypothesis (PPH0) indicates “No association with either trait.” The second hypothesis, posterior probabilities of hypotheses (PPH1), represents, in this gene segment, there is only an association with trait 1 but not with trait 2. Lastly, the third hypothesis (PPH2) signifies “Association with trait 2, but not with trait 1.” Our research assessed the posterior probabilities of hypotheses 3 and 4 (PPH3, PPH4) (27). In hypothesis 3, exposure (plasma protein) and outcome (MN) are linked to the genomic region through different SNPs. In hypothesis 4, the protein and MN are connected to the genomic region through a shared SNP. By employing the coloc.abf algorithm, we defined genes as having gene-based colocalization evidence when PPH4 > 50%.

### 2.8 Phenoscanner

We also employed Phenoscanner to uncover the connections of identified pQTLs with other traits. SNPs were classified as having horizontal pleiotropy when they exhibited connections with established risk factors for MN and plasma protein, encompassing metabolic traits, proteins, or clinical characteristics. For instance, the use of specific medications such as gold compounds, d-penicillamine, and bucillamine, as well as the presence of hepatitis B and liver cancer, which are considered risk factors for MN, were excluded before conducting further analysis.

### 2.9 Mediation analysis

Our two-step analysis unveiled multiple factors that function as mediators in the causal pathway. The overall influence of exposure on the outcomes was categorized into direct and indirect effects (28). Specifically, the total effects of the identified plasma proteins on MN in this study encompassed two components: 1) the direct effects of the identified plasma proteins on MN, calculated through primary MR analysis, and 2) using the product method to estimate the indirect effects mediated by the identified risk factors. Standard errors (SE) and confidence intervals (CI) were determined by employing the delta method. Furthermore, we performed within-group Mendelian randomization (MR) analysis on the plasma proteins included in our study to investigate potential interrelationships that could impact the outcomes of the MR analysis.

### 2.10 Protein-protein interaction network

In the plasma analysis, we investigated proteins potentially associated with MN. We primarily employed MR analysis with a significance level of FDR < 0.10 and directional tests. Furthermore, we explored the target proteins of drugs for MN available in the market. We identified these targeted drugs through a comprehensive literature review and by searching the DrugBank database (DrugBank | Powering health insights with structured drug data) (29, 30). We also searched for drugs targeting the potential pathogenic proteins we identified. Subsequently, protein-protein interaction (PPI) networks were employed to investigate the interplay among the proteins and the relevant targets. All PPI analyses were executed utilizing version 12.0 of the STRING database [STRING: functional protein association networks (string-db. org)] (31), with a predefined minimum threshold set at 0.5.

## 3 Results

### 3.1 Mendelian randomization analysis

**Figure 3:**
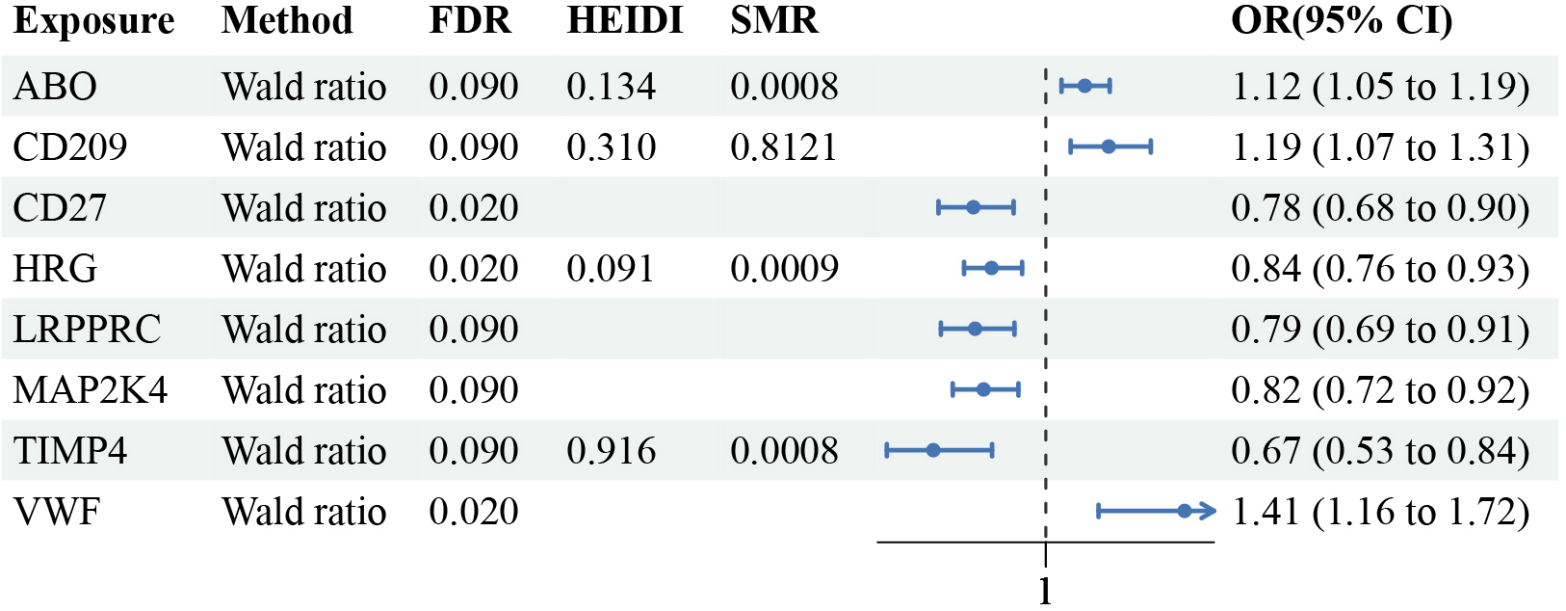
Mendelian randomization results of causal plasma proteins. CI, confidence interval; OR, odds ratio; FDR, p-value after false discovery rate.

Following the adherence to the principles of instrumental variable selection, a comprehensive set of 1056 IVs was included in the analysis (**Supplementary Table 3**). After False Discovery Rate multiple correction, employing a nominal significance threshold (P<0.10) (32–34), the MR analysis identified 8 protein-MN associations. We discovered plasma proteins associated with an increased risk of MN: ABO [(Histo-Blood Group Abo System Transferase) (WR OR = 1.12, 95%CI:1.05-1.19, FDR=0.09, PPH4 = 0.79)], VWF [(Von Willebrand Factor) (WR OR = 1.41, 95%CI:1.16-1.72, FDR=0.02, PPH4 = 0.81)] and CD209 [(Cd209 Antigen) (WR OR = 1.19, 95%CI:1.07-1.31, FDR=0.09, PPH4 = 0.78)]. Proteins that have a protective effect on MN: HRG [(Histidine-Rich Glycoprotein) (WR OR = 0.84, 95%CI:0.76-0.93, FDR=0.02, PPH4 = 0.80)], CD27 [(Cd27 Antigen) (WR OR = 0.78, 95%CI:0.68-0.90, FDR=0.02, PPH4 = 0.80)], LRPPRC [(Leucine-Rich Ppr Motif-Containing Protein, Mitochondrial) (WR OR = 0.79, 95%CI:0.69-0.91, FDR=0.09, PPH4 = 0.80)], TIMP4 [(Metalloproteinase Inhibitor 4) (WR OR = 0.67, 95%CI:0.53-0.84, FDR=0.09, PPH4 = 0.79)] and MAP2K4 [(Dual Specificity Mitogen-Activated Protein Kinase Kinase 4) (WR OR = 0.82, 95%CI:0.72-0.92, FDR=0.09, PPH4 = 0.80)] (**Figure 3**; **Supplementary Table 4**).

### 3.2 Sensitivity analysis and reverse causality detection for MN causal proteins

During the Phenoscanner process, no confounding factors were found (**Supplementary Table 5**). The preliminary study did not detect heterogeneity or horizontal pleiotropy in the analyzed proteins. In the reverse causal analysis (with MN as the exposure and plasma proteins as the outcome), the positive proteins did not exhibit a causal relationship with membranous nephropathy (**Supplementary Tables 6-9**). In addition, we resorted to directional testing to evaluate causality. Furthermore, Steiger filtering provided additional assurance regarding the presence of directional abnormalities. Steiger filtering consistently showed the correct directionality, and the significance level was P<0.05. During the HEIDI test, ABO, HRG, and TIMP4 successfully passed, whereas the remaining five proteins either had missing data or failed to pass the test (**Figure 2**; **Supplementary Table 10**).

### 3.3 Bayesian co-localization analysis

**Figure 4:**
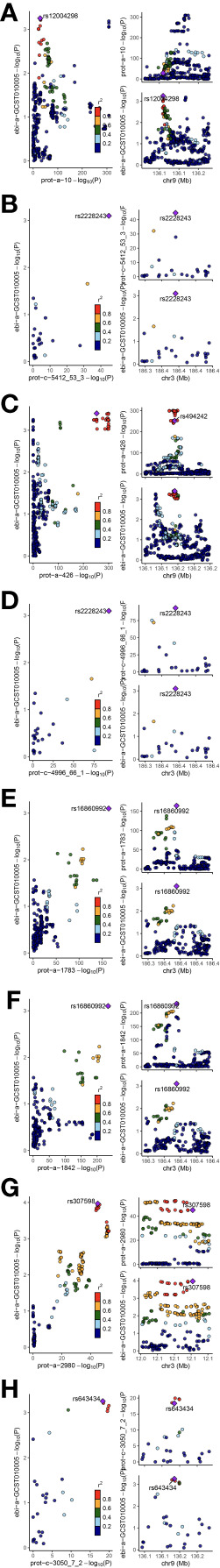
The results of colocalization analysis. **(A)**: ABO; **(B)**: CD209; **(C)**: CD27; **(D)**: HRG; **(E)**: LRPPRC; **(F)**: MAP2K4; **(G)**: TIMP4; **(H)**: VWF. Our findings identified significant protein as having moderate and strong colocalization significance with membranous nephropathy. The figure displays meaningful colocalization results, with the instrumental variables and their chromosomal positions visualized.

Through colocalization analysis, all significant proteins exhibit co-localization with moderate or higher strength [strong Bayesian colocalization (PPH4>0.8) and Moderate-strength Bayesian colocalization (0.5<PPH4≤0.8)]. This indicates these significant plasma proteins share the same variants with MN (**Figure 4**; **Supplementary Table 11**).

### 3.4 The correlation among causal proteins and current drug targets for MN

The PPI network unveiled an interrelationship involving three proteins (CD27) and an existing drug target for MN (CD20 targeted by rituximab). Using STRING, the identified reliable protein interaction axes include MS4A1-CD27-NCAM1, and MS4A1-NCAM1-CD27 (where NCAM1 has been previously confirmed as a pathogenic antigen for MN) (**Supplementary Figure 1**). Therefore, we should pay more attention to the relationship between MS4A1 and CD27. CD27 is linked to the B lymphocyte antigen CD20 (MS4A1) for unknown proteins, which serves as the focus for rituximab. Furthermore, STRING analysis unveiled physical interactions between the proteins above and MS4A1, suggesting their proximity without necessarily being in direct contact. The phenotype scan revealed that the aforementioned correlated proteins remained unaffected by other phenotypes, as all the phenotypes examined were unrelated to MN (**Supplementary Table 12**).

### 3.5 Mediation analysis

Our two-step analysis unveiled multiple factors that function as mediators in the causal pathway. These include adult-onset asthma, beef intake, diabetes or endocrine disease, E-selectin levels, trunk fat, type 1 diabetes with other specified/multiple/unspecified complications, and type 1 diabetes, all of which have the potential to act as mediators in the associations between proteins and diseases (**Supplementary Table 13**). No causal relationships were observed among the various significant proteins.

## 4 Discussion

To our knowledge, this study represents the first attempt to integrate plasma proteomics data with MR and Bayesian colocalization approaches using many databases to investigate the potential involvement of specific proteins in MN. Based on genetic prediction, ABO, CD209, CD27, HRG, LRPPRC, VWF, MAP2K4, and TIMP4 were found to have a causal relationship with MN. Through a series of analyses, we ultimately identified ABO, HRG, and TIMP4 as high-priority potential drug targets for MN. Although CD27, CD209, VWF, LRPPRC, and MAP2K4 had a lower priority, they still hold suggestive significance. Our research in proteomics holds significance, as proteins, acting as effectors in numerous biological processes (35), are widely regarded as drug targets, unlike previous studies that primarily focus on single phenotypes or even precise dual-disease MR, which are typically gene-centric analyses susceptible to the influence of gene-gene and gene-environment interactions. Taking advantage of the rapid advancement in high-throughput sequencing, we integrated genome-wide association studies (GWAS) with proteomics, enabling the integration of genetic and protein-level information. The researchers identified genotype-protein associations (pQTL) that offer novel insights into the genetic control of protein regulation. In the GWAS analysis, overlaps were observed between protein quantitative trait loci (pQTL), gene expression quantitative trait loci (eQTL), and disease-associated loci, indicating the molecular effects of genetic, protein, and disease-associated variations. Further investigations suggest that assuming overlaps between protein quantitative trait loci, gene expression quantitative trait loci, and disease-associated loci, evidence of a causal role of protein biomarkers in disease can be revealed through Mendelian randomization analysis (11). We utilized cis and trans pQTLs as IVs as they are implicated in regulating gene expression at both the transcriptional and translational levels. Cis-pQTLs refer to variants located proximal to the gene encoding the protein under study, while trans-pQTLs represent distal regulatory elements that influence proteins through often unknown mechanisms (36, 37). Trans-pQTLs offer valuable insights into molecular connections in human biology. Trans-pQTLs that overlap with disease associations can identify candidate proteins that were previously not suspected, indicating that genetic loci may influence disease risk through these proteins (11).

By utilizing MR and employing several validation methods, we have newly identified several promising proteins, thereby augmenting their potential as targets for therapeutic interventions. Hence, to identify innovative targets for drug intervention in MN, we utilized a comprehensive analysis to evaluate the causal involvement of proteins in MN. Causal relationships identified through MR may include reverse causality, horizontal pleiotropy, or genetic confounding induced by linkage disequilibrium (LD). Therefore, in this study, we adhered to the three fundamental principles of instrumental variable selection in Mendelian randomization analysis. We utilized IVs that exhibited strong correlations with plasma proteins. We excluded IVs that demonstrated linkage disequilibrium during the implementation of MR. The reverse Mendelian randomization analysis revealed no reverse causal relationship between these proteins and MN.

Additionally, Bayesian colocalization was employed to eliminate bias introduced by LD (24). Using a posterior probability threshold of 0.5, it was considered to have moderate to high co-localization strength, and we identified 8 proteins that may share the same MN variants. Nevertheless, these associations alone do not completely explain the connection between the identified proteins and MN. Fortunately, Phenoscanner analysis unveiled associations of the IVs for these proteins with various phenotypes such as Duodenal ulcer, APTT, and thrombosis. These diverse phenotype associations are unrelated to MN, making it unlikely that the results are biased. Therefore, ABO, CD209, CD27, HRG, LRPPRC, VWF, MAP2K4, and TIMP4 may serve as potential drug targets for MN, with particular emphasis on ABO, HRG and TIMP4. We also conducted network and database searches for targeted drugs against CD27, such as MK-5890 (monoclonal antibody) (38), Varlilumab (monoclonal antibody) (39), and CDX-527 (bispecific antibody) (40). These drugs hold promise as novel targeted therapies for the treatment of MN.

CD20 is not found as a plasma protein in our database. However, CD20, an antigen present in B cells, is a key target in treating membranous nephropathy (MN) using rituximab (RTX), a chimeric monoclonal antibody. It should be noted that CD20 may not meet the criteria as the circulating biomarker under consideration in our analysis. RTX, besides its application in MN, demonstrates effectiveness in treating various kidney diseases like minimal change disease (MCD), membranous glomerulonephritis (MGN), and lupus nephritis (LN). Notably, reducing B cell-mediated antibody production is a well-known protective mechanism in antibody-mediated kidney diseases. By impeding the formation of antigen-antibody complexes, RTX achieves its therapeutic effects.

First, epigenetics regulates gene expression levels and affects protein synthesis and function, while post-transcriptional modification refers to the chemical modification of RNA molecules after RNA is transcribed. In MN, the expression levels of some genes may change, affecting glomerular structure and function. In this study, significant proteins are molecules of great interest in MN, closely related to immune regulation and inflammatory processes. In terms of epigenetics, significant protein gene expression may be regulated by DNA methylation and histone modifications. Methylation is a common DNA epigenetic modification that can affect the transcriptional activity of genes. Histone modifications involve the interaction between histone proteins and DNA, such as acetylation, methylation, and ubiquitination. These modifications may regulate the expression level of the significant protein gene and affect the function and activity of significant proteins in immune cells. In terms of post-transcriptional modification, significant protein mRNA molecules may be subject to a variety of chemical modifications, such as splicing, RNA modification, and RNA degradation. These modifications can affect the stability and post-transcriptional regulation of significant protein mRNA, thereby affecting the synthesis and function of significant proteins. For example, mRNA splicing can produce different forms of the significant protein transcript, thereby affecting its post-translational protein structure and function. An in-depth study of the epigenetic and post-transcriptional modification mechanisms of significant proteins will help understand the pathogenesis of membranous nephropathy and provide a theoretical basis for the development of related treatment strategies.

Furthermore, the genetic impact on the abundance of plasma proteins is generally governed by mRNA regulation, although this influence is not entirely definitive. It may also encompass genetic effects on processes beyond transcription, such as protein degradation, binding, secretion, or clearance from circulation. When the above-mentioned situation occurs, it may result in data loss during pQTL analysis using the HEIDI test.

ABO, Histo-blood group ABO system transferase is a critical protein involved in determining the ABO blood group system, which encompasses three carbohydrate antigens: A, B, and H. This protein facilitates the transfer of specific sugar molecules onto the surfaces of red blood cells, thereby determining an individual’s blood type within the ABO system. This may suggest that blood type has an impact on the risk of developing MN.

CD209, CD209 antigen, and the pathogen-recognition receptor are expressed on the surface of immature dendritic cells (DCs) and play a crucial role in initiating the primary immune response. It is believed to mediate the endocytosis of pathogens, which are then degraded in lysosomal compartments. Subsequently, the receptor returns to the cell membrane surface, presenting pathogen-derived antigens to resting T-cells through MHC class II proteins, thereby initiating the adaptive immune response.

CD27 functions as a receptor for CD70/CD27L and may play a crucial role in the survival of activated T-cells. The MR result suggests that for every one standard deviation increase in circulating levels of CD27, there is a 22% decrease in the risk of MN. It is implicated in cellular apoptosis through its interaction with SIVA1 (41). CD27 exhibits protein-protein interactions with CD20 and NCAM1. While CD20-targeted therapy, rituximab, has become the mainstream treatment for MN. The combination of PPI network analysis and MR revealed that therapeutic agents targeting CD27 have exhibited promising progress and evaluation in clinical trials, in contrast to the other identified targets in our study. Currently, targeted drugs against CD27, such as MK-5890 (monoclonal antibody), Varlilumab (monoclonal antibody), and CDX-527 (bispecific antibody), hold potential as new targeted therapies for the treatment of MN. Based on our research findings, we hypothesize that these antibodies may have an impact on MN, and therefore, we recommend future clinical studies to investigate this hypothesis further.

HRG is also known as Histidine-Rich Glycoprotein. Plasma glycoprotein can bind to various ligands, such as hemoglobin, acetylated hepari and plasminogen. After FDR correction, it still remains significant. The MR result suggests that for every one standard deviation increase in circulating levels of HRG, there is a 16% increase in the risk of MN.

LRPPRC, The leucine-rich PPR motif-containing protein is involved in RNA metabolism in both the nucleus and mitochondria. In the nucleus, it binds to poly(A) mRNAs and is associated with mRNA maturation and export. In mitochondria, it binds to poly(A) mRNA and affects the translation and stability of cytochrome c oxidase subunits.

VWF, also known as Von Willebrand Factor. It plays a vital role in hemostasis and serves as a carrier for clotting factors. Upon blood vessel damage, a significant number of platelets adhere to collagen fibers, forming a clot, where VWF acts as a mediator in hemostasis. The binding of VWF factor to clotting factor VIII stabilizes and regulates its activity. After FDR correction, it still remains significant. The MR result suggests that for every one standard deviation increase in circulating levels of VWF, there is a 41% decrease in the risk of MN.

Dual specificity mitogen-activated protein kinase kinase 4 (MAP2K4) is a protein kinase that plays a crucial role in the MAP kinase signal transduction pathway, specifically in the stress-activated protein kinase/c-Jun N-terminal kinase (SAP/JNK) signaling pathway. It directly activates MAPK8/JNK1, MAPK9/JNK2, and MAPK10/JNK3, making it one of the few known kinases to do so.

Metalloproteinase inhibitor 4 (TIMP-4) forms complexes with metalloproteinases, specifically collagenases, and inactivates their activity by binding to the catalytic zinc cofactor.

In conclusion, we utilized proteomics and MR analysis to identify drug targets for MN. Unlike previous studies that focused solely on the genetic level, our analysis incorporated both genetic and protein levels. Considering the limited availability of targeted drugs for the treatment of MN, we have successfully identified several potential therapeutic targets that require further investigation in the future.

There are several limitations in our research. Firstly, the plasma protein data we utilized was obtained from a combination of multiple proteomic studies, which may employ varying standards for protein measurement. Nevertheless, we selected data that matched circulating protein data with GWAS. Secondly, most proteins are associated with only one SNP, indicating either a lack of trans-pQTL or cis-pQTL. Thirdly, the data we employed in our experiments exclusively represented European populations, necessitating further research to generalize the findings to other ethnicities. Moreover, no proteins other than CD27 were found to be associated with CD20 in the protein-protein interaction (PPI) network. This indicates that these proteins may function independently of CD20 and serve as additional potential targets. We attempted to explore the relationship between significant proteins and the targets affected by RTX monoclonal antibody using PPI analysis. However, the precise mechanisms underlying this interaction remain unknown to us. Lastly, it is crucial to acknowledge that unvalidated data analysis may include confounding factors that require further investigation through experimental validation. Our data can be utilized for future research in establishing the validation of drug targets. A more profound comprehension of the genetic regulation of protein drug targets and the circulating levels of biomarkers has the potential to improve drug interventions and clinical trials.

## Data availability statement

Plasma pQTL data was derived via public research (15), MN data was retrieved from Kiryluk Lab (20). We used R language version 4.3.0 for our analysis. In R language, we utilized the “coloc (https://github.com/chr1swallace/coloc.git)” and “TwoSampleMR (https://github.com/MRCIEU/TwoSampleMR.git)” and “locuscompare” packages for our analysis (42).

## Ethics statement

The data utilized in this analysis were obtained from publicly accessible databases. All of the data have been de-identified and received ethical approval from the appropriate ethics committee. Consequently, this study does not necessitate individual ethical approval.

## Author contributions

ZS: Conceptualization, Data curation, Formal analysis, Funding acquisition, Investigation, Methodology, Project administration, Resources, Software, Supervision, Validation, Visualization, Writing – original draft, Writing – review & editing. QW: Conceptualization, Funding acquisition, Project administration, Resources, Supervision, Validation, Visualization.

## Supplementary material

Supplementary Table 1 STROBE-MR checklist table.

Supplementary Table 2 Database exposure.

Supplementary Table 3 Instrumental variables for MR analysis.

Supplementary Table 4 MR analysis results.

Supplementary Table 5 Confounding factors.

Supplementary Table 6 Instrumental variables for reverse MR analysis.

Supplementary Table 7 Heterogeneity analysis results.

Supplementary Table 8 Horizontal pleiotropy results.

Supplementary Table 9 Reverse MR analysis results.

Supplementary Table 10 HEIDI test results.

Supplementary Table 11 Co-localization analysis results.

Supplementary Table 12 PPI network results.

Supplementary Table 13 Mediation analysis results and data.

Supplementary Figure 1 PPI network visualization.
