# Supplementary material for: Potential therapeutic targets for membranous nephropathy: proteome-wide Mendelian randomization and colocalization analysis
Source: Front Immunol. 2024 Apr 19;15:1342912. doi: 10.3389/fimmu.2024.1342912 (PMC11069303; doi:10.3389/fimmu.2024.1342912)
Supplement: Supplementary Table 1 — STROBE-MR checklist table. [file Table_1.docx]

**STROBE-MR checklist of recommended items to address in reports of Mendelian randomization studies**^1^ ^2^

| **Item No.** | **Section** | **Checklist item** | **Relevant text from manuscript** |
| --- | --- | --- | --- |
| 1 | **TITLE and ABSTRACT** | Indicate Mendelian randomization (MR) as the study’s design in the title and/or the abstract if that is a main purpose of the study | Potential therapeutic targets for Membranous Nephropathy: proteome-wide Mendelian randomization and colocalization analysis |
|  | **INTRODUCTION** |  |  |
| 2 | **Background** | Explain the scientific background and rationale for the reported study. What is the exposure? Is a potential causal relationship between exposure and outcome plausible? Justify why MR is a helpful method to address the study question | The application of Mendelian randomization (MR) analysis has recently gained significant traction in identifying potential therapeutic targets and repurposing drugs(8). As an approach within genetic instrumental variable analysis, MR employs single nucleotide polymorphisms (SNPs) as instrumental variables (IVs) to estimate the causal relationship between exposures and outcomes(9). Compared to traditional observational studies, MR can mitigate the impacts of type I errors and confounding factors(10). |
| 3 | **Objectives** | State specific objectives clearly, including pre-specified causal hypotheses (if any). State that MR is a method that, under specific assumptions, intends to estimate causal effects | The application of Mendelian randomization (MR) analysis has recently gained significant traction in identifying potential therapeutic targets and repurposing drugs(8). As an approach within genetic instrumental variable analysis, MR employs single nucleotide polymorphisms (SNPs) as instrumental variables (IVs) to estimate the causal relationship between exposures and outcomes(9). Compared to traditional observational studies, MR can mitigate the impacts of type I errors and confounding factors(10). |
|  | **METHODS** |  |  |
| 4 | **Study design and data sources** | Present key elements of the study design early in the article. Consider including a table listing sources of data for all phases of the study. For each data source contributing to the analysis, describe the following: | Plasma protein quantitative trait loci dataGenome-wide association study data of Membranous nephropathy |
|  | a) | Setting: Describe the study design and the underlying population, if possible. Describe the setting, locations, and relevant dates, including periods of recruitment, exposure, follow-up, and data collection, when available. |  |
|  | b) | Participants: Give the eligibility criteria, and the sources and methods of selection of participants. Report the sample size, and whether any power or sample size calculations were carried out prior to the main analysis |  |
|  | c) | Describe measurement, quality control and selection of genetic variants |  |
|  | d) | For each exposure, outcome, and other relevant variables, describe methods of assessment and diagnostic criteria for diseases |  |
|  | e) | Provide details of ethics committee approval and participant informed consent, if relevant |  |
| 5 | **Assumptions** | Explicitly state the three core IV assumptions for the main analysis (relevance, independence and exclusion restriction) as well assumptions for any additional or sensitivity analysis | Selection of Instrumental Variables |
| 6 | **Statistical methods: main analysis** | Describe statistical methods and statistics used | Mendelian randomization analysisSelection of Instrumental Variables |
|  | a) | Describe how quantitative variables were handled in the analyses (i.e., scale, units, model) |  |
|  | b) | Describe how genetic variants were handled in the analyses and, if applicable, how their weights were selected |  |
|  | c) | Describe the MR estimator (e.g. two-stage least squares, Wald ratio) and related statistics. Detail the included covariates and, in case of two-sample MR, whether the same covariate set was used for adjustment in the two samples |  |
|  | d) | Explain how missing data were addressed |  |
|  | e) | If applicable, indicate how multiple testing was addressed |  |
| 7 | **Assessment of assumptions** | Describe any methods or prior knowledge used to assess the assumptions or justify their validity | Selection of Instrumental Variables |
| 8 | **Sensitivity analyses and additional analyses** | Describe any sensitivity analyses or additional analyses performed (e.g. comparison of effect estimates from different approaches, independent replication, bias analytic techniques, validation of instruments, simulations) | HEIDI test |
| 9 | **Software and pre-registration** |  | Data availability |
|  | a) | Name statistical software and package(s), including version and settings used |  |
|  | b) | State whether the study protocol and details were pre-registered (as well as when and where) |  |
|  | **RESULTS** |  |  |
| 10 | **Descriptive data** |  | Following the adherence to the principles of instrumental variable selection, a comprehensive set of 1057 IVs was included in the analysis. After False Discovery Rate multiple correction, employing a nominal significance threshold (P＜0.10)(32–34), the MR analysis identified 8 protein-MN associations. |
|  | a) | Report the numbers of individuals at each stage of included studies and reasons for exclusion. Consider use of a flow diagram |  |
|  | b) | Report summary statistics for phenotypic exposure(s), outcome(s), and other relevant variables (e.g. means, SDs, proportions) |  |
|  | c) | If the data sources include meta-analyses of previous studies, provide the assessments of heterogeneity across these studies |  |
|  | d) | For two-sample MR:  i.  Provide justification of the similarity of the genetic variant-exposure associations between the exposure and outcome samples  ii.  Provide information on the number of individuals who overlap between the exposure and outcome studies | Plasma protein and MN come from separate cohorts, indicating no sample overlap between the databases. This suggests that the probability of bias occurring is 0, and the likelihood of a Type I error is 5% (https://sb452.shinyapps.io/overlap/)(21). |
| 11 | **Main results** |  | Selection of Instrumental Variables; After False Discovery Rate multiple correction, employing a nominal significance threshold (P＜0.10)(32–34), the MR analysis identified 8 protein-MN associations. We discovered plasma proteins associated with an increased risk of MN: ABO [ (Histo-Blood Group Abo System Transferase) (WR OR = 1.12, 95%CI：1.05-1.19, FDR=0.09, PPH4 = 0.79)], VWF [(Von Willebrand Factor) (WR OR = 1.41, 95%CI：1.16-1.72, FDR=0.02, PPH4 = 0.81)] and CD209 [ (Cd209 Antigen) (WR OR = 1.19, 95%CI：1.07-1.31, FDR=0.09, PPH4 = 0.78)]. Proteins that have a protective effect on MN: HRG [(Histidine-Rich Glycoprotein) (WR OR = 0.84, 95%CI：0.76-0.93, FDR=0.02, PPH4 = 0.80)], CD27 [(Cd27 Antigen) (WR OR = 0.78, 95%CI：0.68-0.90, FDR=0.02, PPH4 = 0.80)], LRPPRC [(Leucine-Rich Ppr Motif-Containing Protein, Mitochondrial) (WR OR = 0.79, 95%CI：0.69-0.91, FDR=0.09, PPH4 = 0.80)], TIMP4 [(Metalloproteinase Inhibitor 4) (WR OR = 0.67, 95%CI：0.53-0.84, FDR=0.09, PPH4 = 0.79)] and MAP2K4 [(Dual Specificity Mitogen-Activated Protein Kinase Kinase 4) (WR OR = 0.82, 95%CI：0.72-0.92, FDR=0.09, PPH4 = 0.80)] (Figure 2). |
|  | a) | Report the associations between genetic variant and exposure, and between genetic variant and outcome, preferably on an interpretable scale |  |
|  | b) | Report MR estimates of the relationship between exposure and outcome, and the measures of uncertainty from the MR analysis, on an interpretable scale, such as odds ratio or relative risk per SD difference |  |
|  | c) | If relevant, consider translating estimates of relative risk into absolute risk for a meaningful time period |  |
|  | d) | Consider plots to visualize results (e.g. forest plot, scatterplot of associations between genetic variants and outcome versus between genetic variants and exposure) |  |
| 12 | **Assessment of assumptions** |  | During the HEIDI test, ABO, HRG, and TIMP4 successfully passed, whereas the remaining five proteins either had missing data or failed to pass the test (Figure 2). |
|  | a) | Report the assessment of the validity of the assumptions |  |
|  | b) | Report any additional statistics (e.g., assessments of heterogeneity across genetic variants, such as *I^2^*, Q statistic or E-value) |  |
| 13 | **Sensitivity analyses and additional analyses** |  | During the HEIDI test, ABO, HRG, and TIMP4 successfully passed, whereas the remaining five proteins either had missing data or failed to pass the test (Figure 2). |
|  | a) | Report any sensitivity analyses to assess the robustness of the main results to violations of the assumptions |  |
|  | b) | Report results from other sensitivity analyses or additional analyses | Bayesian co-localization analysisThe correlation among causal proteins and current drug targets for MNMediation analysis |
|  | c) | Report any assessment of direction of causal relationship (e.g., bidirectional MR) | In the reverse causal analysis (with MN as the exposure and plasma proteins as the outcome), the positive proteins did not exhibit a causal relationship with membranous nephropathy. In addition, we resorted to directional testing to evaluate causality. Furthermore, Steiger filtering provided additional assurance regarding the presence of directional abnormalities. Steiger filtering consistently showed the correct directionality, and the significance level was P＜0.05. |
|  | d) | When relevant, report and compare with estimates from non-MR analyses |  |
|  | e) | Consider additional plots to visualize results (e.g., leave-one-out analyses) |  |
|  | **DISCUSSION** |  |  |
| 14 | **Key results** | Summarize key results with reference to study objectives | To our knowledge, this study represents the first attempt to integrate plasma proteomics data with MR and Bayesian colocalization approaches using many databases to investigate the potential involvement of specific proteins in MN. Based on genetic prediction, ABO, CD209, CD27, HRG, LRPPRC, VWF, MAP2K4, and TIMP4 were found to have a causal relationship with MN. Through a series of analyses, we ultimately identified ABO, HRG, and TIMP4 as high-priority potential drug targets for MN. Although CD27, CD209, VWF, LRPPRC, and MAP2K4 had a lower priority, they still hold suggestive significance. |
| 15 | **Limitations** | Discuss limitations of the study, taking into account the validity of the IV assumptions, other sources of potential bias, and imprecision. Discuss both direction and magnitude of any potential bias and any efforts to address them | There are several limitations in our research. Firstly, the plasma protein data we utilized was obtained from a combination of multiple proteomic studies, which may employ varying standards for protein measurement. Nevertheless, we selected data that matched circulating protein data with GWAS. Secondly, most proteins are associated with only one SNP, indicating either a lack of trans-pQTL or cis-pQTL. Thirdly, the data we employed in our experiments exclusively represented European populations, necessitating further research to generalize the findings to other ethnicities. Moreover, no proteins other than CD27 were found to be associated with CD20 in the protein-protein interaction (PPI) network. This indicates that these proteins may function independently of CD20 and serve as additional potential targets. We attempted to explore the relationship between significant proteins and the targets affected by RTX monoclonal antibody using PPI analysis. However, the precise mechanisms underlying this interaction remain unknown to us. Lastly, it is crucial to acknowledge that unvalidated data analysis may include confounding factors that require further investigation through experimental validation. Our data can be utilized for future research in establishing the validation of drug targets. A more profound comprehension of the genetic regulation of protein drug targets and the circulating levels of biomarkers has the potential to improve drug interventions and clinical trials. |
| 16 | **Interpretation** |  | Discussion |
|  | a) | Meaning: Give a cautious overall interpretation of results in the context of their limitations and in comparison with other studies |  |
|  | b) | Mechanism: Discuss underlying biological mechanisms that could drive a potential causal relationship between the investigated exposure and the outcome, and whether the gene-environment equivalence assumption is reasonable. Use causal language carefully, clarifying that IV estimates may provide causal effects only under certain assumptions |  |
|  | c) | Clinical relevance: Discuss whether the results have clinical or public policy relevance, and to what extent they inform effect sizes of possible interventions |  |
| 17 | **Generalizability** | Discuss the generalizability of the study results (a) to other populations, (b) across other exposure periods/timings, and (c) across other levels of exposure | Thirdly, the data we employed in our experiments exclusively represented European populations, necessitating further research to generalize the findings to other ethnicities. |
|  | **OTHER INFORMATION** |  |  |
| 18 | **Funding** | Describe sources of funding and the role of funders in the present study and, if applicable, sources of funding for the databases and original study or studies on which the present study is based | Shenzhen Key Medical Discipline Construction Fund (SZXK009) and SZSM202211013. |
| 19 | **Data and data sharing** | Provide the data used to perform all analyses or report where and how the data can be accessed, and reference these sources in the article. Provide the statistical code needed to reproduce the results in the article, or report whether the code is publicly accessible and if so, where | Plasma pQTL data was derived via public research(15), MN data was retrieved from Kiryluk Lab(20). |
| 20 | **Conflicts of Interest** | All authors should declare all potential conflicts of interest | The authors declare that the research was conducted in the absence of any commercial or financial relationships that could be construed as a potential conflict of interest. |

This checklist is copyrighted by the Equator Network under the Creative Commons Attribution 3.0 Unported (CC BY 3.0) license.

1. Skrivankova VW, Richmond RC, Woolf BAR, Yarmolinsky J, Davies NM, Swanson SA, et al. Strengthening the Reporting of Observational Studies in Epidemiology using Mendelian Randomization (STROBE-MR) Statement. JAMA. 2021;under review.

2. Skrivankova VW, Richmond RC, Woolf BAR, Davies NM, Swanson SA, VanderWeele TJ, et al. Strengthening the Reporting of Observational Studies in Epidemiology using Mendelian Randomisation (STROBE-MR): Explanation and Elaboration. BMJ. 2021;375:n2233.
