# Supplementary figures and images for: Potential therapeutic targets for membranous nephropathy: proteome-wide Mendelian randomization and colocalization analysis
Source: Front Immunol. 2024 Apr 19;15:1342912. doi: 10.3389/fimmu.2024.1342912 (PMC11069303; doi:10.3389/fimmu.2024.1342912)

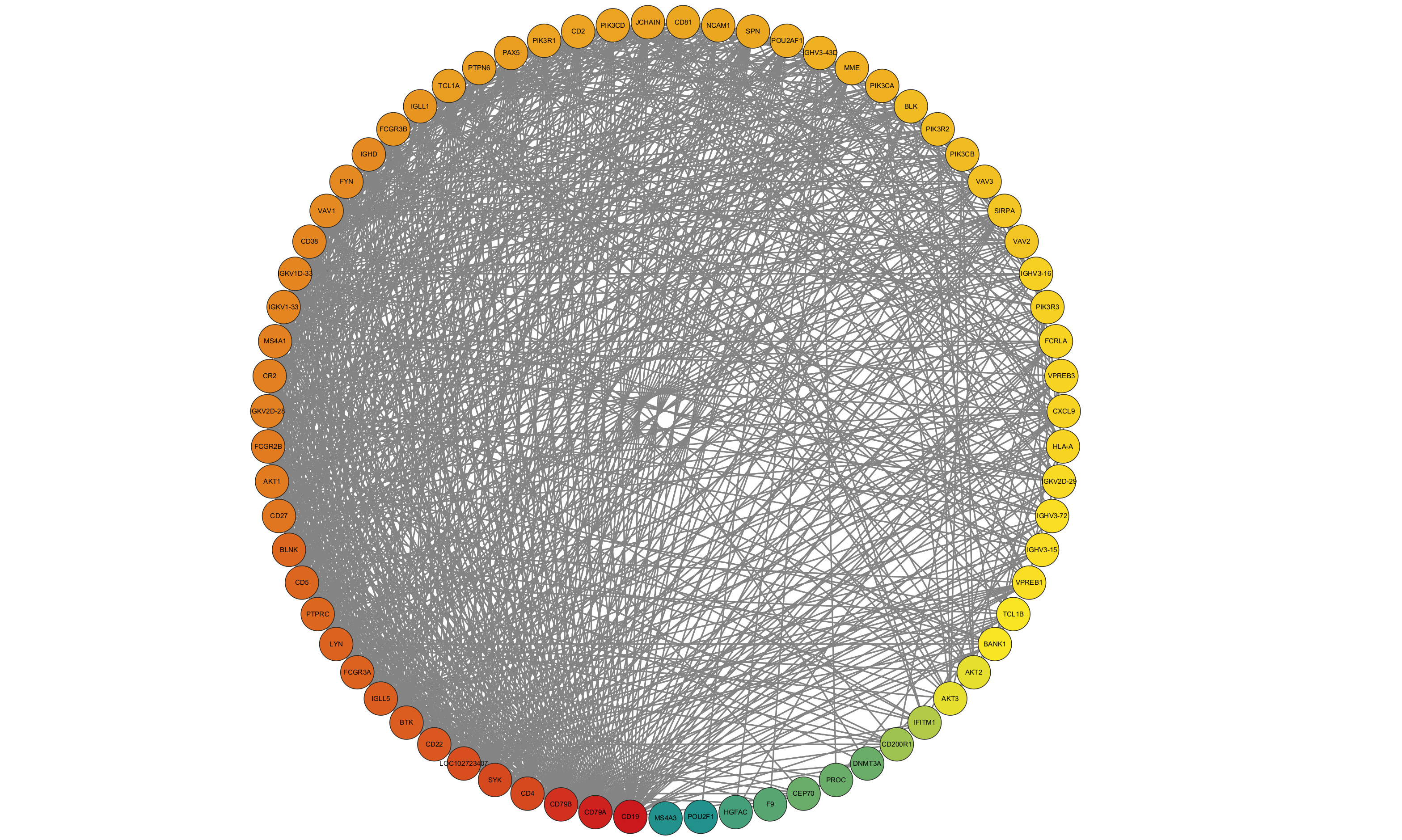

Supplement: Supplementary Figure 1 — PPI network visualization. [file Image_1.png]
